# Appearance of necrotizing sialometaplasia temporarily associated with MDMA consumption

**DOI:** 10.4317/jced.63836

**Published:** 2026-02-26

**Authors:** Eymi Plaza-Matos, José López-Vicente, Nerea Larrea-Rodríguez, Javier Alberdi-Navarro

**Affiliations:** 1 DDS. Department of Stomatology. School of Medicine and Nursing. University of the Basque Country (EHU). Leioa, Spain; 2 DDS, MD, PhD. Department of Stomatology. School of Medicine and Nursing. University of the Basque Country (EHU). Leioa, Spain; 3 DDS, PhD. Department of Stomatology. School of Medicine and Nursing. University of the Basque Country (EHU). Leioa, Spain; 4 DDS, MS Oral Pathology, PhD. Department of Stomatology. School of Medicine and Nursing. University of the Basque Country (EHU). Leioa, Spain

## Abstract

We reported the case of a 24-year-old woman presented with necrotizing sialometaplasia associated temporally with 3,4-methylenedioxymethamphetamine (MDMA) use. The patient reported having consumed MDMA powder licked onto the oral mucosa days before the appearance of the lesions. Symptomatic treatment of the presenting lesions was carried out, with a follow-up that demonstrated complete healing of the lesions. The clinical case is presented and possible pathophysiological mechanisms that may justify the relationship between MDMA and necrotizing sialometaplasia are discussed. This report describes the relationship between a new, previously undescribed aetiological factor related to necrotizing sialometaplasia.

## Introduction

Necrotizing sialometaplasia (NS) is a benign, non-neoplastic inflammatory condition, first described by Abrams et al., in 1973. It is characterized by necrosis of the salivary gland acinar parenchyma and squamous metaplasia of the ductal epithelium (1). In 2022, necrotizing sialometaplasia was included in the section on non-neoplastic lesions in the fifth edition of the World Health Organization (WHO) Classification of Head and Neck Tumors book (2). NS is a self-limiting pathology that is clinically characterized, in most cases, by a unilateral ulcerated lesion on the palate, although in 17% of cases it presents bilaterally (3 , 4). Occasionally, ulceration may be preceded by localized swelling. Although it can occur at any age, this pathology most commonly affects adults in their fifth decade of life (3 , 4). The etiopathogenic factors are not well understood; however, histological studies suggest that the condition is caused by ischemia of the minor salivary glands (5 , 6). Some possible factors thought to promote ischemia include traumatic lesions, anesthetic infiltration, dentures, alcohol consumption, smoking, radiation exposure, allergies, respiratory conditions, previous adenoidectomy, adjacent tumors, and eating disorders, particularly bulimia (3 - 6). Among these factors, the use of certain substances, such as tobacco, alcohol and cocaine, has been reported to be associated with the development of NS (6). It is currently unknown whether these factors act individually or synergistically, as well as the nature of their interrelationship. Additionally, the role of tissue hypoxia in the early stages of NS and the secretion of TGF-3 by fibroblasts have recently been described as important pathophysiological mechanisms associated with the development of NS (7 , 8). Clinically, NS is characterized by the appearance of a rapidly evolving ulcerative lesion on the palate (3). This often requires performing a biopsy in a significant number of cases to confirm the clinical diagnosis and to rule out other pathologies, particularly those of a malignant nature (3 , 5). The ulcerated presentation and rapid progression may mimic malignant or infectious pathologies, potentially leading to unnecessary treatment (2 , 3). Despite its clinical presentation, NS is a benign and self-limiting condition that typically heals by secondary intention within 3 to 12 weeks without the need for treatment (3 - 6). Given the ongoing controversy regarding the possible etiopathogenic factors associated with NS, we present a clinical case in which the patient temporally linked the onset of symptoms with the use of multiple substances, including 3,4-methylenedioxymethamphetamine (MDMA), and discuss its potential role in the development of NS.

## Case Report

**Figure 1:**
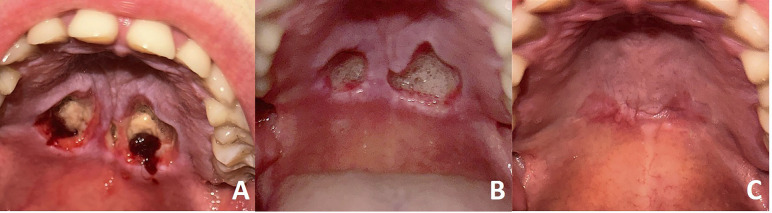
Clinical image (A). Inicial presentation. Ulcerated lesions at the posterior hard palate. (B). 2 weeks follow-up. Significant improvement of the lesions. (C). 8 weeks follow-up. Complete healing of the lesions.

A 24-year-old woman was referred to the dental clinic with painful lesions on the hard palate. The patient reported having noticed the lesions, which had evolved over a few days. She described occasional prior use of cocaine and MDMA, which she associated with the onset of the lesions. Moreover, she reported smoking approximately 15 marijuana joints daily. Relevant medical history included a diagnosis of bulimia nervosa, for which she was undergoing treatment with olanzapine and escitalopram. It is noteworthy that the patient reported no episodes of vomiting for several weeks prior to the appearance of the lesions. She also reported being allergic to penicillin. Clinical examination revealed two ulcerated lesions located bilaterally on the posterior hard palate. The lesions were measured between 1.5-2 cm, had a fibrinoid, necrotic base and presented well-defined, non-indurated borders. Blood clots could be observed inside the lesion, and the hard palate showed hyperkeratinization (Fig. 1A).

1

Based on these findings, a presumptive clinical diagnosis of necrotizing sialometaplasia was made. This diagnosis was based on several clinical aspects, including the rapid evolution of the clinical picture, the location of the lesions on the palate, as well as the presence of bilateral lesions, the non-elevated edges of the ulcerated lesions, and the temporal relationship with related ischemic factors. Symptomatic treatment with chlorhexidine gel (0.20%) combined with hyaluronic acid (0.20%), applied three times daily, was initiated. The lesions were re-evaluated after two weeks, showing significant improvement but without complete healing (Fig. 1B). Consequently, symptomatic treatment was maintained, and regular follow-ups were conducted until complete healing was confirmed after eight weeks of monitoring (Fig. 1C). The patient was referred to the psychiatric service to receive specific treatment for her drug addiction, as well as to control her bulimia nervosa. We kept her under observation for a year, with no recurrence of the lesions.

## Discussion

Necrotizing sialometaplasia is a rare, benign, self-limiting condition whose etiology and pathogenesis remain poorly understood. The most widely accepted cause is ischemia of the minor salivary glands, typically leading to necrosis and subsequent deep mucosal ulceration (1 , 3 , 5). This ulceration generally heals spontaneously but is accompanied by symptoms such as pain and altered sensation, presenting the healing process is progressive but slow (4). Among the ischemia-related factors relevant to this case are bulimia (3 , 5 , 6) and the use of vasoconstrictive substances such as cocaine (9) and tobacco (4). Nevertheless, MDMA has not previously been described as an etiological factor of NS. MDMA is a recreational drug chemically related to amphetamines. Among its known effects, it has been shown to induce severe vasoconstriction (10). Given this, it is plausible that MDMA use could promote ischemia of the palatal mucosa and infarction of the minor salivary glands located in the area. In addition, other tissue effects associated with MDMA use may favor the appearance of NS, including the generation of reactive oxygen species and activation of matrix metalloproteinases (MMPs), particularly MMP-9 and MMP-3 (11). Furthermore, MDMA has been shown to promote ischemic events by enhancing hemostasis and coagulation (10 , 11). MDMA is commonly available in tablet or powder form, and can be administered via ingestion, intranasal insufflation (snorting) or direct contact with the oral mucosa (e.g., licking). In this case, the patient reported consuming MDMA in powder form by licking it. It is important to note that MDMA acts as a vasoconstrictor at the site of application, and oral mucosal lesions related to its use have been documented (12 , 13). Such lesions typically appear 24-48h after drug administration (12), consistent with the timeline observed in this case. Other oral effects associated with MDMA use include xerostomia, bruxism and dental erosion, which seem to correlate with dose and frequency of use (12). Therefore, a possible association between MDMA consumption and the development of NS is suggested, although further studies are necessary to confirm this hypothesis. The decision to implement topical treatment was based on chlorhexidine's ability, as an antiseptic, to reduce the possibility of superinfection of ulcerated lesions (14). Hyaluronic acid is a compound that accelerates the healing of the oral mucosa. In fact, the combination of both compounds has been widely used in other clinical conditions involving ulceration with bone exposure, such as cases of oral ulceration and bone sequestration (15). The use of these active ingredients was empirical. The absence of previous studies and the fact that this is a clinical description prevents us from knowing the possible role they may have played in the healing/curing time of the lesions. Regarding clinical management of the lesions, symptomatic treatment was initiated, and the patient was re-evaluated after two weeks, at which point improvement in their clinical appearance was observed. Had there been no improvement after two weeks, a biopsy would have been indicated to exclude other conditions, including malignant neoplasms or infectious diseases (1 , 3). Although the absence of an incisional biopsy and its histopathological evaluation may be considered a limitation of the case presented, we believe that this fact is justified by the evolution of the case itself. Although no recurrence of the existing lesions was observed during the follow-up period, it is important to highlight the importance of controlling the pathologies or factors that contribute to the onset of this condition. In our case, we consider that cessation of drug use and treatment of bulimia are fundamental to the therapeutic approach. This is relevant, as it is expected that if the pathophysiological factors that may be involved in the onset of NS persist, the clinical condition may be recurrent. In conclusion, NS may be associated temporally with MDMA use, but this possible relationship requires further investigation to support the hypothesis presented in this case. A comprehensive evaluation of all potential etiopathogenic factors is essential in NS cases to better understand the causes of lesion development.

## Data Availability

The datasets used and/or analyzed during the current study are available from the corresponding author.
